# Protective Effects of Chinese Traditional Medicine Buyang Huanwu Decoction on Myocardial Injury

**DOI:** 10.1093/ecam/nep013

**Published:** 2011-06-05

**Authors:** Guangde Yang, Zhiyuan Fang, Yu Liu, Hui Zhang, Xiaolian Shi, Qiaoli Ji, Qinqin Lin, Rong Lin

**Affiliations:** ^1^Department of Pharmacology and Pharmacy, Key Laboratory of Environment and Genes Related to Diseases, Xi'an Jiaotong University, School of Medicine, Xi'an, Shaanxi 710061, China; ^2^Shaanxi Provincial People's Hospital, Xi'an, Shaanxi 710068, China

## Abstract

Many clinical studies have reported that Buyang Huanwu Decoction (BYHWD) has a protective effect on ischemic heart disease (IHD). In the present study, the protective effect of BYHWD on myocardial ischemia was investigated. Different doses of BYHWD and Compound Danshen Dropping Pills (CDDP) were lavaged to rats, respectively, isoproterenol (ISO) was intraperitoneally injected in to all animals to induce myocardial ischemia except the control group. Electrocardiogram (ECG) of each animal was recorded; activities of lactate dehydrogenase (LDH), creatine kinase (CK) and aspartate aminotransferase (AST) in serum were detected. As the results of ECG showed, pre-treatment with BYHWD inhibited ischemic myocardial injury, and the activities of LDH, CK and AST were lower than those in the myocardial ischemia model group, which suggests that BYHWD rescues the myocardium from ischemia status. To research the potential mechanism, the level of nitric oxide (NO), nitric oxide syntheses (NOS) and inducible nitric oxide syntheses (iNOS), the expression of iNOS and ligand of cluster of differentiation 40 (CD40L) were detected. The results revealed that BYHWD significantly decreased the level of NO, NOS and iNOS in serum. Moreover, BYHWD decreased the expression of iNOS and CD40L in myocardial tissues. These results indicate that the protective effect of BYHWD on myocardial ischemia and mechanism are associated with inhibition of iNOS and CD40L expression.

## 1. Introduction

Nitric oxide (NO) is a reactive nitrogen species produced by nitric oxide syntheses (NOS). Basal generation of NO has physiological and homeostatic effects, but high level of NO production is often associated with pathological conditions [1]. A number of cellular constituents of cardiac muscle, including the cardiac myocytes, are known to be capable of expressing inducible nitric oxide syntheses (iNOS) in response to stimuli [2]. Increasing data support the idea that iNOS plays a deleterious role in the setting of myocardial ischemia and infarction [3, 4].

In recent years, inflammation has been recognized as a major force driving ischemic process. Inflammatory cytokines may damage the vascular endothelial surface directly and lead to ischemia [5]. Increasing evidence showing that increased level of inflammatory markers is related to ischemic heart disease (IHD) [6]. Furthermore, several inflammatory markers have been used to assess cardiovascular risks, which affirms that inflammation is a predictor of cardiovascular events recently [7]. In the past decade, the cluster of differentiation 40 (CD40) and its ligand (CD40L), as a major factor in chronic inflammatory and autoimmune diseases [8], attracted more and more interest of the medical community. Some studies demonstrated that high level of CD40L was associated with increased risk of cardiovascular events in patients with acute coronary syndrome. Moreover, healthy women with high levels of CD40L have been suggested to at an increased risk of cardiovascular events [9].

In China, many formulas and extracts can be used to treat IHD, Compound Danshen Dropping Pills (CDDP) is the first formula affirmed by US Food and Drug Administration for its excellent effect on IHD [10]. Buyang Huanwu Decoction (BYHWD), a well-known traditional Chinese medicine formula for activating blood circulation to dissipate blood stasis, has been used for improving neurological functional recovery in stroke-induced disability in China for centuries [11]. Some other studies demonstrated that BYHWD could down-regulate the expression of iNOS after cerebral ischemia in mice [12]. Recently, some studies have reported that BYHWD can be used to treat ischemic coronary heart disease by relieving angina pectoris [13]. However little is known about the exact mechanism. In the present study, the protective effect of BYHWD on myocardial injury induced by isoproterenol (ISO) in rat was investigated by measuring the activities of lactate dehydrogenase (LDH), creatine kinase (CK) and aspartate aminotransferase (AST) in serum. To further investigate the potential mechanism, the level of NO, NOS and iNOS in serum, the expression of iNOS and CD40L in myocardial tissues of this animal model were detected.

## 2. Materials and Methods

Trihydroxymethyl aminomethane (Tris) glycine and sodium dodecyl sulfate (SDS) were purchased from Amresco (Amresco, USA). Anti-iNOS antibody was purchased from Beijing Zhongshan Jinqiao Biology Corporation (P.R. China). Anti-CD40L antibody was purchased from Wuhan Boster Biology Engineering Corporation (Wuhan, P.R. China). LDH, CK, AST, NO, NOS and iNOS assay kits were produced by the Institute of Nanjing Jiancheng Biology Engineering (Nanjing, P.R. China). Protein extraction reagent (RIPA) kit was purchased from Bioteke Corporation (Beijing, P.R. China). ISO was purchased from Sigma Biotechnology (Sigma, USA). CDDP was obtained from Tianshili Pharmaceuticals Company (Tianjin, P.R. China). All other agents used were of the commercially available grade.

### 2.1. Preparation of BYHWD

BYHWD consists of seven medicinal components, including Milkvetch Root, Chinese Angelica, Szechwan Lovage Rhizome, Red Peony Root, Earth Worm, Peach Seed and Safflower, the components were mixed in order with the ratio of 120 : 6 : 3 : 4.5 : 3 : 3 : 3 (dry weight). All of the herbal components were originally purchased from Xi'an Pharmacy of Beijing Tongrentang, and they were stated above with GAP grade. The drugs were extracted with standard methods according to Chinese Pharmacopoeia (China Pharmacopoeia and Committee, 2000). These crude drugs were soaked in distilled water and boiled for 30 min twice, and the drug solution was filtered through a mesh, then the filtrate was concentrated to 3 g mL^−1^ by a vacuum pump and stored at 4°C until use. CDDP was used here as a positive control drug.

### 2.2. Experimental Animals and Model

Sixty male adult Sprague-Dawley (SD) rats weighing 180–200 g were provided by the Medical Experimental Animal Center of Xi'an Jiaotong University (Xi'an, P.R. China). The rats were divided into six groups randomly: control group, model group, CDDP group (0.073 g kg^−1^/day) and three BYHWD groups (25.680, 12.840 and 6.420 g kg^−1^/day). When the experiment began, BYHWD and CDDP were lavaged to the animals once a day while the control group and the model group were lavaged with saline for 14 days. From the 12th day, all animals except those in the control group received ISO (0.03 g kg^−1^/day) intraperitoneal injection simultaneously once a day for 3 days, while the control group was injected with saline. At the end of the experiment, animals were anesthetized with intrapentobarbital sodium (0.04 g kg^−1^/day) by peritoneal injection. After electrocardiogram (ECG) was recorded, blood samples were collected by cardiac puncture and heart tissue samples were excised parallel to coronary sulcus, 3 mm apart from cardiac apex. All samples were frozen in liquid nitrogen immediately for further examinations. The experimental protocol was in accordance with the National Institutes of Health Guide for Care and Use of Laboratory Animals and was approved by the Institutional Animal Care Committee of Xi'an Jiaotong University.

### 2.3. Determination of the Activities of LDH, CK and AST

In order to evaluate the extent of heart injury induced by ISO, the activities of LDH, CK and AST in serum of rats were measured. When animals were killed, blood sample was collected and centrifuged at 800 rpm for 5 min, and then the serum was collected. The activity of LDH, CK and AST were detected by pyruvic acid method, *N*-broncholysin method and malate dehydrogenase method, respectively. All the assay kits were commercially available and all operations followed the instructions of the kits. The findings were detected on an ultraviolet/visible scanning spectrophotometer.

### 2.4. Determination of the Levels of NO, NOS and iNOS

The serum was collected for detection of NO, NOS and iNOS levels. NO level in serum was detected by nitrate reductase method while NOS and iNOS level in serum was detected by chromatometry. All the assay kits were commercially available and all operations followed the instructions of the kits. The findings were detected on an ultraviolet/visible scanning spectrophotometer.

### 2.5. Western Blotting

To investigate the potential mechanism, the expression of iNOS in myocardial tissues was detected by Western blotting. The total protein of myocardial tissues was extracted following the instruction of RIPA and protein concentration was quantified with a BCA protein quantity assay kit. The same amounts of protein was separated by 10% SDS-polyacrylamide gel electrophoresis (SDS-PAGE) at 4°C, and then the protein was transferred onto microporous polyvinylidene fluoride membranes in running buffer with 20% methanol. After nonspecific sites were blocked with 5% milk-Tris-buffered saline-tween 20 (TBST), membranes were incubated with anti-iNOS antibody (1 : 400) and anti-*β*-actin antibody (1 : 400) at 4°C overnight, respectively. Then, membranes were washed with TBST and a horseradish peroxidase-linked antibody was employed as a secondary antibody (1 : 1000), the bands of interest were detected using an enhanced chemiluminescent technique. Densities of bands were measured by an image analyzer.

### 2.6. Immunohistochemistry

The myocardial tissues samples were separated from the heart, and were fixed in 10% phosphate-buffered formalin and embedded in paraffin and 5 *μ*m sections were prepared. For detection of CD40L expression, sections were incubated with 0.1 M citrate buffer (pH 6.0) for 30 min at 95°C for antigen retrieval and quenching endogenous peroxidase; then the sections were incubated with horse serum blocking solution for 10 min and treated with rabbit anti-CD40L polyclonal antibody (1 : 100) for 2 h at room temperature. After being washed with 0.01 M PBS containing 0.05% Tween-20, the sections were incubated with a biotinylated secondary IgG antibody, and then the sections were treated with streptavidin peroxidase and aminoethyl carbazole. Finally, the sections were visualized with Olympus BX51 microscopy, and the number of CD40L-positive stained cells in myocardium was counted at a magnification of 200x and the ratio of CD40L-positive stained cells/total myocardial cell was calculated.

### 2.7. Statistical Analysis

Data are presented as means ± SD. Statistical analysis was carried out with three or more groups using one-way analysis of variance (ANOVA) and Dunnetts' test. The values of *P* < .05 were considered statistically significant.

## 3. Results

### 3.1. Myocardial Protective Effect on ECG Change in ISO-Induced Ischemia

**Figure 1:**
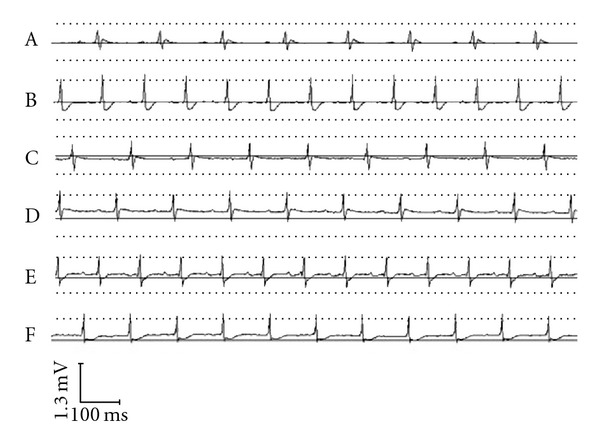
Myocardial protective influence on ECG change in ISO-induced ischemia: A myocardial ischemia animal model was established by giving ISO. CDDP and BYHWD were administered to the animals. ECG of the animals was recorded. (A) Control group. (B) Model group. (C) CDDP group (0.073 g kg^−1^/day). (D) BYHWD group (25.680 g kg^−1^/day). (E) BYHWD group (12.840 g kg^−1^/day). (F) BYHWD group (6.420 g kg^−1^/day).

In order to investigate whether BYHWD could protect the myocardium from ischemia, ECG of the animals was recorded by an electrocardiogram recorder. In the model group, the ECG presented an obvious myocardial ischemia with a more than 0.l mV of descending at the ST segment. Pre-treatment with BYHWD (25.680, 12.840 and 6.420 g kg^−1^/day) and CDDP (0.073 g kg^−1^/day) significantly improved the change of ECG by attenuating the descending of the ST segment (Figure 1).

### 3.2. BYHWD Inhibited the Increase of LDH, CK and AST Activities in Serum

**Figure 2:**
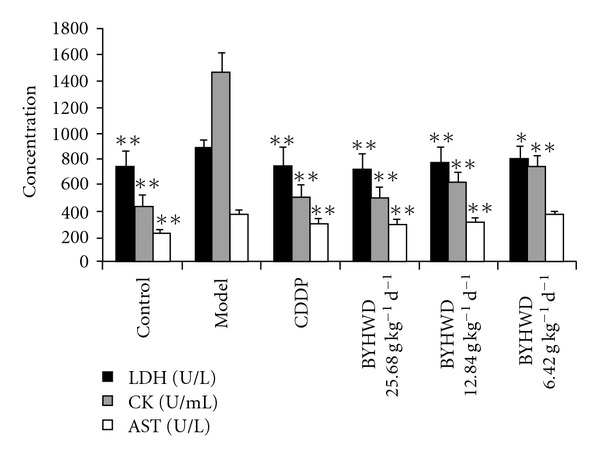
BYHWD inhibits the increase of LDH, CK and AST activities in serum: A myocardial ischemia animal model was established by giving ISO. CDDP and BYHWD were administered to the animals. The serum concentration of LDH, CK and AST were detected. Results were presented as means ± SD; *P < .05, **P < .01, compared with those in the model group.

The activities of LDH, CK and AST were increased significantly in the model group compared with those in the control group (*P * < .01). Pre-treatment with BYHWD (25.680, 12.840 and 6.420 g kg^−1^/day) and CDDP (0.073 g kg^−1^/day) could decrease the activities of these enzymes in serum. The AST activity in BYHWD of 25.680 and 12.840 g kg^−1^/day and CDDP groups was significantly less than the model group, respectively (*P * < .01), and the LDH and CK activities in all drug-used groups were also less than those in the model group, respectively (CK, *P* < .01; LDH, *P* < .01 or *P* < .05) (Figure 2).

### 3.3. BYHWD Inhibited the Increase of NO, NOS and iNOS Levels in Serum

**Figure 3:**
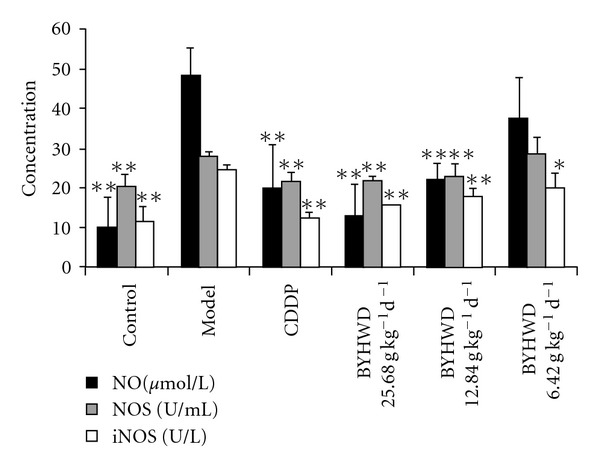
BYHWD inhibits the increase of NO, NOS and iNOS levels in serum: A myocardial ischemia animal model was established by giving ISO. CDDP and BYHWD were administered to the animals. The serum concentration of NO, NOS and iNOS were detected by chromatometry. Results were presented as means ± SD, *P < .05, **P < .01, compared with those in the model group.

To determine the potential mechanism by which BYHWD protects the myocardium from ischemia, the levels of NO, NOS and iNOS in serum were detected. The results showed that the levels of NO, NOS and iNOS in serum were significantly increased in the model group compared with the control group (*P * < .01), but, significantly deceased by pre-treatment of BYHWD (25.680, 12.840 and 6.420 g kg^−1^/day) and CDDP (0.073 g kg^−1^/day). Compared with that in the model group, there was a significantly difference in the level of iNOS in all BYHWD and CDDP used groups (*P * < .01 or *P * < .05); the differences in the levels of NO and NOS were also significant except the BYHWD 6.420 g kg^−1^/day group (*P * < .01) (Figure 3).

### 3.4. BYHWD Decreased the iNOS Expression in Myocardial Tissues

**Figure 4:**
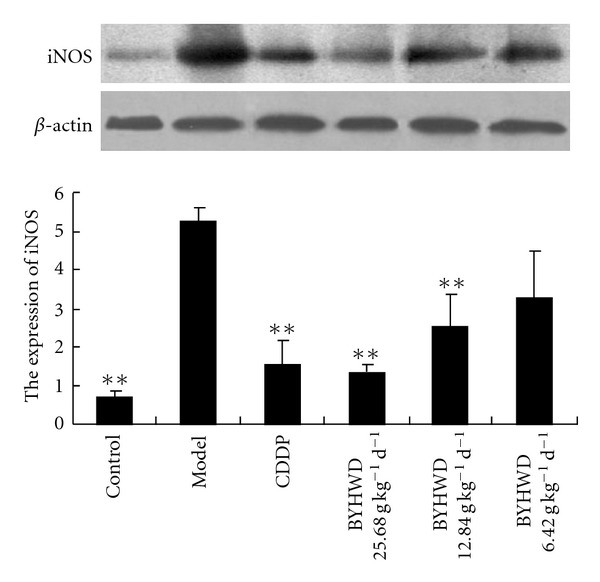
BYHWD decreases the iNOS expression in myocardial tissues: A myocardial ischemia animal model was established by giving ISO. CDDP and BYHWD were administered to the animals. The expression of iNOS in myocardial tissues was detected by Western blotting. Results were presented as means ± SD. *P < .05, **P < .01, compared with those in the model group.

As the results showed, there was few expression of iNOS protein in the control group whereas it was significantly increased in the model group (*P * < .01). Pre-treatment with BYHWD (25.680, 12.840 and 6.420 g kg^−1^/day) and CDDP (0.073 g kg^−1^/day) could decrease the expression of iNOS in a dose-dependent manner. Compared with that in the model group, the iNOS protein expression was significantly decreased (*P * < .01) in all BYHWD and CDDP used groups with an exception of the BYHWD 6.420 g kg^−1^/day group (Figure 4).

### 3.5. BYHWD Decreased the CD40L Expression in Myocardial Tissues

**Figure 5:**
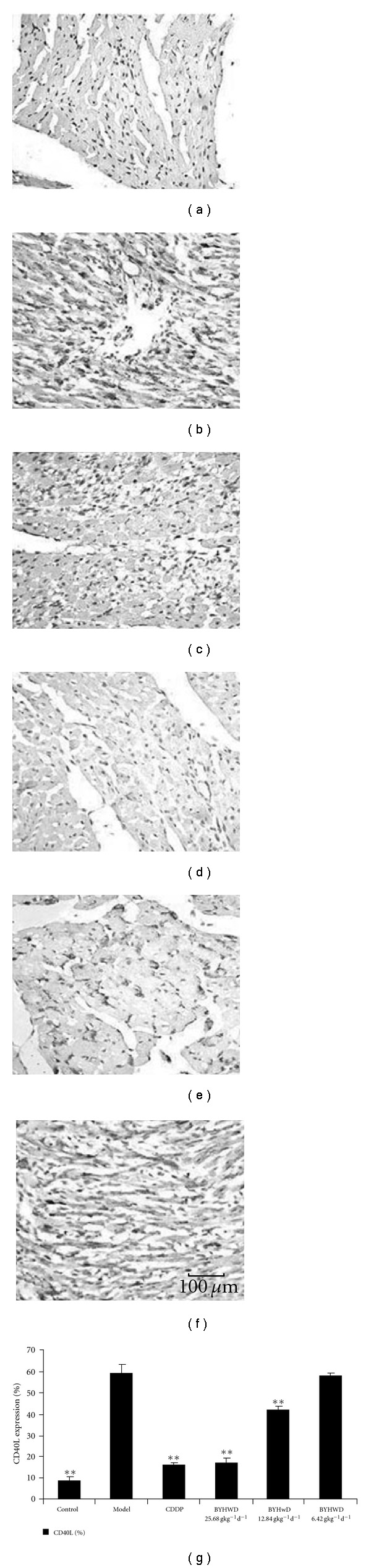
BYHWD decreases the CD40L expression in myocardial tissues (Bar = 100 *μ*m): A myocardial ischemia animal model was established by giving ISO. CDDP and BYHWD were administered to the animals. The expression of CD40L in myocardial tissues was detected by immunohistochemistry, and then the ratio of CD40L-positive stained cells/total myocardial cell was calculated. (a) Control group. (b) Model group. (c) CDDP group (0.073 g kg^−1^/day). (d) BYHWD group (25.680 g kg^−1^/day). (e) BYHWD group (12.840 g kg^−1^/day). (f) BYHWD group (6.420 g kg^−1^/day).

As shown in Figure 5, a few CD40L-positive stained cells were found in control myocardial tissues. However, the number of CD40L-positive stained cells was remarkably increased in the model group (*P <* .01). Similarly, plenty of cells were CD40L-positive stained in BYHWD 6.420 g kg^−1^/day group and there was no difference in the ratio of CD40L-positive stained cells/total myocardial cells between these two groups. In contrast, the number of CD40L-positive stained cells in the other groups was decreased significantly and there was a statistically significant difference in the ratio of CD40L-positive stained cells/total myocardial cells compared with the model group (*P* < .01).

## 4. Discussion

BYHWD is a classic traditional Chinese medicine formula has been widely used for therapy of ischemic cerebral disease, such as cerebral ischemia, brain infarction and stroke-induced disability in clinic [14, 15]. Some studies indicated that the effects of BYHWD on ischemic cerebral disease were attributed to its capacity of increasing cerebral blood flow, decreasing infarction volume, attenuating injury of cerebral blood vessels and cerebral edema, reducing neural apoptosis [16, 17]. In recent years, some clinical studies have reported that BYHWD had the capacity of relieving angina pectoris, improving myocardial ischemia, decreasing the volume of myocardial infarction, suggesting that BYHWD can be used to the therapy of IHD [18, 19].

In the present study, the protective effect of BYHWD on myocardial ischemia was investigated by measuring the change of ECG, as well as the activities of LDH, CK and AST in serum in a myocardial ischemic animal model induced by ISO. Usually, ECG is a useful parameter for determination of myocardial ischemia in clinic and experiment. There are many changes of ECG after myocardial ischemia, including amplitude of R wave, prolonging of QRS duration, elevating or descending of ST segment and change of T wave [20, 21]. The descending of ST segment reflects insufficiency of acute coronary circulation or non-symptomatic myocardial ischemia [22]. In present study, we found that the ST segment descended after ISO injection in the model group while the use of BYHWD previously attenuated this change, suggesting that BYHW prevented myocardial ischemia induced by ISO in rats. To further study the protective effect of BYHWD on myocardial ischemia, the activities of LDH, CK and AST in serum were detected. LDH, CK and AST are mainly present in cytoplasmia of myocardium and play an important role in myocardial tissue energy metabolism [23]. CK is an important enzyme for aerobic metabolism of cells by catalyzing the conversion of creatine to phosphocreatine. LDH is a key enzyme for glycolysis that catalyzes the interconversion of lactate and pyruvate. AST can catalyze the reversible transfer of an amino group from aspartate to (alpha)-ketoglutarate to form glutamate and oxaloacetate [24]. When the myocardium is injured, these enzymes can be released from the cells to serum, the activities of these enzymes in serum reflect the extent of myocardium injury. Therefore, the activities of these enzymes in serum are sensitive biomarkers for heart injury [20]. As shown in the results, the activities of these enzymes were significantly increased in the model group, but pre-treatment with BYHWD could significantly decrease the LDH, CK and AST activities in serum, suggesting that BYHWD can prevent the myocardium injury induced by ISO in rats.

NO is a major regulator for cardiovascular system by regulating basal vascular tone, blood pressure and tissue perfusion [25]. There are three mammalian isoforms of NOS: neuronal NOS (nNOS), inducible NOS (iNOS) and endothelial NOS (eNOS). NO is usually synthesized by endothelial nitric oxide synthase (eNOS) in endothelium, but it also can be produced abundantly by iNOS which is induced by cytokines and lipopolysaccharide, ischemia, stroke, trauma, and infection [26]. eNOS and nNOS isoenzymes are constitutive and calcium-calmodulin-dependent enzymes. The former is expressed in the endothelium under basal conditions and responsible for the endothelium-dependent vasodilatation [26], while the latter is expressed in central nervous system and responsible for the mediation of the metabolism/blood flow coupling into the brain. iNOS is not constitutively expressed in heart, but it can be induced by pathological conditions [27]. Once induced, iNOS is capable of producing large amounts of NO at 100 times greater than normal by resident cardiac cells and activated immune cells that infiltrate to the injured heart [28, 29]. Overexpression of iNOS contributes to myocardial dysfunction and higher mortality after myocardial infarction, resulting in peroxynitrite generation, heart block and sudden death [28], while inhibition of iNOS improves heart function in myocardial infarction and reduces infarct size [29, 30]. A recent study indicates that chronic *β*-adrenergic receptor stimulation can upregulate iNOS expression and increase NO production in the heart, which subsequently markedly enhances formation of reactive nitrogen species/peroxynitrite in the heart, thereby eliciting myocardial apoptosis and potentiating myocardial ischemia/reperfusion injury [31]. In accordance with these studies, we found that the levels of NO and iNOS in serum and the expression of iNOS protein in myocardial tissues were increased significantly after ISO injection in the model group, which implies that NO and iNOS participated in the development of ischemic myocardial injury. Using gene knockout animals, increased NO production from iNOS expression is reported to contribute to myocardial dysfunction and mortality after myocardial infarction in mice [32]. Selective and continuous inhibition of iNOS can exert protective effects with respect to myocardial performance, coronary blood flow, cellular infiltration and reduction of infarct size [3]. In previous studies, it was demonstrated that BYHWD could improve cerebral ischemia via down-regulating the expression of iNOS in mice, implying that iNOS downregulating is a potential mechanism by which BYHWD improves ischemic diseases [12]. In the present study, we also found that BYHWD pre-treatment could significantly decrease the levels of NO and iNOS in serum and the iNOS expression in myocardial tissues compared with those in the model group, suggesting that BYHWD can decrease the NO level in serum by down-regulating the expression of iNOS after myocardial ischemia induced by ISO.

Increasing evidence supports that inflammation plays an important role in IHD, including myocardial ischemia or silent myocardial ischemia [33], and acute coronary syndromes [34]. Anti-inflammatory therapy can modify the disease process in IHD significantly [35]. Many studies indicate that CD40L plays a role in IHD, and increase of level of soluble CD40L is a predictor of cardiovascular events in general populations [36]. In addition, the interaction of CD40/CD40L plays a dominant role in stimulating of NO production in murine peritoneal macrophages [37]. Inhibiting the CD40/CD40L interaction may significantly decrease NO production in CD40L gene knockout mice [38]. To further understand the mechanism by which BYHWD protects myocardial tissues from ischemia, the expression of CD40L in myocardial tissues were examined. The results showed that the expression of CD40L was increased significantly in myocardial tissues of the model group, while pre-treatment with BYHWD can significantly inhibit the expression of CD40L, suggesting that anti-inflammation by decreasing the CD40L expression is one of the potential mechanisms by which BYHWD protects myocardium from ischemia in rats.

In conclusion, our results suggest that BYHWD has a protective effect against myocardial ischemia induced by ISO in rats, and BYHWD inhibition of the iNOS and CD40L expression in myocardial tissues is the potential mechanism of this protective action. These findings provide a pharmacological basis for the clinical application of traditional Chinese medicine BYHWD in prevention of ischemic cardiovascular disease. However, post-treatment with this decoction whether also could produce a treating effect on myocardial ischemia is little known. It requires further study.

## Funding

This work was supported by the National Natural Science Foundation of China (No. 90709018), and the Key Science Research Project of Xi'an, Shaanxi Province (2006GG06174).

## References

[1] Jugdutt BI (2002). Nitric oxide and cardioprotection during ischemia-reperfusion. *Heart Failure Reviews*.

[2] Balligand J-L, Ungureanu D, Kelly RA (1993). Abnormal contractile function due to induction of nitric oxide synthesis in rat cardiac myocytes follows exposure to activated macrophage-conditioned medium. *The Journal of Clinical Investigation*.

[3] Wildhirt SM, Weismueller S, Schulze C, Conrad N, Kornberg A, Reichart B (1999). Inducible nitric oxide synthase activation after ischemia/reperfusion contributes to myocardial dysfunction and extent of infarct size in rabbits: evidence for a late phase of nitric oxide-mediated reperfusion injury. *Cardiovascular Research*.

[4] Arstall MA, Sawyer DB, Fukazawa R, Kelly RA (1999). Cytokine-mediated apoptosis in cardiac myocytes: the role of inducible nitric oxide synthase induction and peroxynitrite generation. *Circulation Research*.

[5] Shah PK (2000). Circulating markers of inflammation for vascular risk prediction: are they ready for prime time. *Circulation*.

[6] Lombardi F, Tundo F, Terranova P (2005). Prognostic value of C-reactive protein in patients with stress induced myocardial ischemia. *International Journal of Cardiology*.

[7] Armstrong EJ, Morrow DA, Sabatine MS (2006). Inflammatory biomarkers in acute coronary syndromes—part II: acute-phase reactants and biomarkers of endothelial cell activation. *Circulation*.

[8] Lutgens E, Daemen MJAP (2002). CD40-CD40L interactions in atherosclerosis. *Trends in Cardiovascular Medicine*.

[9] Tanne D, Haim M, Goldbourt U (2006). CD40 ligand and risk of ischemic stroke or coronary events in patients with chronic coronary heart diseaseB. *International Journal of Cardiology*.

[10] Tian Y, Li L, Wang R, Ji L (2005). Compound danshen dropping pills: an overview of its protective effect on heart. *Acta Chinese Medicine and Pharmacology*.

[11] Chen A, Wang H, Zhang J (2008). BYHWD rescues axotomized neurons and promotes functional recovery after spinal cord injury in rats. *Journal of Ethnopharmacology*.

[12] Sun X, Zhao Y, Xie X, Cai Y, Zheng Y (2006). Effect of Buyanghuanwu recipe on the expression of inducible nitric oxide synthase in atherosclerosis mice. *Pharmacology and Clinical Application of Chinese Materia Medica*.

[13] Wei L, Ding S (2002). Therapeutic effect of improved Buyang Huanwu Decoction (decocted in wine) on coronary artery disease. *Journal of Shandong University of Traditional Chinese Medicine*.

[14] Wu Y, Jiang L (2000). Clinical study on influence of Buyang Huanwu Decoction on the metabolic imbalance of endothelin and calcitonin gene related peptide in patients with early cerebral infarction. *Chinese Journal of Integrated Traditional and Western Medicine*.

[15] Zhang H, Liang M, Ma Z, Ye S (1996). Clinical study on effects of Buyang Huanwu Decoction on coronary heart disease. *Chinese Journal of Integrated Traditional and Western Medicine*.

[16] Zhang J, Li C, Guo X, Wang Y (1999). Influence of Buyang Huanwu Decoction on activity of blood platelet activating factor receptor in rabbit. *Chinese Journal of Integrated Traditional and Western Medicine*.

[17] Wang M, Deng C (2000). Overview and prospect of anti-cerebral ischemia research of Buyang Huanwu Decoction. *Journal of Hunan College of Traditional Chinese Medicine*.

[18] Wang J (2006). Treatment of 60 cases of angina with improved Buyang Huanwu Decoction. *Practice of Traditional Chinese Internal Medicine*.

[19] Zeng F (2007). Application of Buyang Huanwu Decoction in cardiovascular and peripheral vascular disease. *Practice of Traditional Chinese Internal Medicine*.

[20] Guo J (2006). Electrocardiogram of acute coronary syndrome. *Journal of Clinical Eletrocardiology*.

[21] Guo J (2006). Electrocardiogram of myocardium reperfusion. *Journal of Clinical Eletrocardiology*.

[22] Huang Z (2005). Clinical meaning of ST Segment descending. *Journal of Clinical Eletrocardiology*.

[23] Xu S (1991). *Experimental Method of Pharmacology*.

[24] Howard-Alpe GM, Sear JW, Foex P (2006). Methods of detecting atherosclerosis in non-cardiac surgical patients; the role of biochemical markers. *British Journal of Anaesthesia*.

[25] Razavi HM, Hamilton JA, Feng Q (2005). Modulation of apoptosis by nitric oxide: implications in myocardial ischemia and heart failure. *Pharmacology and Therapeutics*.

[26] Kelly RA, Balligand J-L, Smith TW (1996). Nitric oxide and cardiac function. *Circulation Research*.

[27] Haywood GA, Tsao PS, von der Leyen HE (1996). Expression of inducible nitric oxide synthase in human heart failure. *Circulation*.

[28] Mungrue IN, Gros R, You X (2002). Cardiomyocyte overexpression of iNOS in mice results in peroxynitrite generation, heart block, and sudden death. *The Journal of Clinical Investigation*.

[29] Li D, Qu Y, Tao L (2006). Inhibition of iNOS protects the aging heart against *β*-adrenergic receptor stimulation-induced cardiac dysfunction and myocardial ischemic injury. *Journal of Surgical Research*.

[30] Saito T, Hu F, Tayara L, Fahas L, Shennib H, Giaid A (2002). Inhibition of NOS II prevents cardiac dysfunction in myocardial infarction and congestive heart failure. *American Journal of Physiology*.

[31] Hu A, Jiao X, Gao E (2006). Chronic beta-adrenergic receptor stimulation induces cardiac apoptosis and aggravates myocardial ischemia/reperfusion injury by provoking inducible nitric-oxide synthase-mediated nitrative stress. *Journal of Pharmacology and Experimental Therapeutics*.

[32] Feng Q, Lu X, Jones DL, Shen J, Arnold JM (2001). Increased inducible nitric oxide synthase expression contributes to myocardial dysfunction and higher mortality after myocardial infarction in mice. *Circulation*.

[33] Li J-J (2004). Silent myocardial ischemia may be related to inflammatory response. *Medical Hypotheses*.

[34] Pasqui AL, Di Renzo M, Bova G (2006). Pro-inflammatory/anti-inflammatory cytokine imbalance in acute coronary syndromes. *Clinical and Experimental Medicine*.

[35] Suzuki J, Ogawa M, Maejima Y (2007). Tea catechins attenuate chronic ventricular remodeling after myocardial ischemia in rats. *Journal of Molecular and Cellular Cardiology*.

[36] Malarstig A, Lindahl B, Wallentin L, Siegbahn A (2006). Soluble CD40L levels are regulated by the -3459 A > G polymorphism and predict myocardial infarction and the efficacy of antithrombotic treatment in non-ST elevation acute coronary syndrome. *Arteriosclerosis, Thrombosis, and Vascular Biology*.

[37] Bingaman AW, Pearson TC, Larsen CP (2000). The role of CD40L in T cell-dependent nitric oxide production by murine macrophages. *Transplant Immunology*.

[38] Al-Ramadi BK, Fernandez-Cabezudo MJ, Ullah A, El-Hasasna H, Flavell RA (2006). CD154 is essential for protective immunity in experimental *Salmonella* infection: evidence for a dual role in innate and adaptive immune responses. *The Journal of Immunology*.

